# Factors associated with COVID-19 Infection among a national population of individuals with schizophrenia or schizoaffective disorder in the United States

**DOI:** 10.1186/s12888-022-04026-7

**Published:** 2022-06-02

**Authors:** Joshua N. Liberman, Jacqueline Pesa, Mary Pat Petrillo, Charles Ruetsch

**Affiliations:** 1 Health Analytics, LLC, Columbia, MD USA; 2 Janssen Scientific Affairs, Titusville, NJ USA

**Keywords:** COVID-19, Schizophrenia, Risk factors, Social determinants

## Abstract

**Background:**

Individuals with schizophrenia are a vulnerable and under-served population who are also at risk for severe morbidity and mortality following COVID-19 infection. Our research was designed to identify factors that put individuals with schizophrenia at increased risk of COVID-19 infection.

**Methods:**

This study was a retrospective cohort analysis of medical and pharmacy claims among 493,796 individuals residing in the United States with schizophrenia or schizoaffective disorder, between January 1, 2019 and June 30, 2020. A confirmed diagnosis of COVID-19 infection by September 30, 2020 was regressed on demographics, social determinants, comorbidity, and pre-pandemic (December 2019 – February 2020) healthcare utilization characteristics.

**Results:**

A total of 35,249 (7.1%) individuals were diagnosed with COVID-19. Elevated odds of COVID-19 infection were associated with age, increasing consistently from 40-49 years (OR: 1.16) to 80+ years (OR:5.92), male sex (OR: 1.08), Medicaid (OR: 2.17) or Medicare (OR: 1.23) insurance, African American race (OR: 1.42), Hispanic ethnicity (OR: 1.23), and higher Charlson Comorbidity Index. Select psychiatric comorbidities (depressive disorder, adjustment disorder, bipolar disorder, anxiety, and sleep-wake disorder) were associated with elevated odds of infection, while alcohol use disorder and PTSD were associated with lower odds. A pre-pandemic psychiatry (OR:0.56) or community mental health center (OR:0.55) visit were associated with lower odds as was antipsychotic treatment with long-acting injectable antipsychotic (OR: 0.72) and oral antipsychotic (OR: 0.62).

**Conclusions:**

Among individuals with schizophrenia, risk of COVID-19 infection was substantially higher among those with fewer economic resources, with greater medical and psychiatric comorbidity burden, and those who resided in African American or Hispanic communities. In contrast, individuals actively engaged in psychiatric treatment had substantially lower likelihood of infection. These results provide insights for healthcare providers that can translate into improved identification of at-risk individuals and interventions to reduce the risk and consequences of COVID-19 infection.

## Introduction

Schizophrenia is characterized by symptoms of psychosis (e.g., hallucinations, delusions, and thought disorder), negative symptoms (e.g., social and emotional withdrawal), and cognitive dysfunction (e.g., difficulties with attention, memory, concentration). Schizophrenia can be a disabling condition, with 50% of those individuals who receive a diagnosis having long-term psychiatric problems and 20% having chronic symptoms and disability [1]. As a result, individuals with schizophrenia are often exposed to high-risk social determinants of health: unemployment, lower socioeconomic status, and lower education levels [2]. As a result, individuals with schizophrenia are a particularly vulnerable and often under-served population [3].

These factors appear to increase both the risk of COVID-19 infection and the risk of severe morbidity and mortality following infection. Recently published research indicates that individuals with any serious mental illness, including schizophrenia, have 1.5 times the risk of COVID-19 infection [4], due at least in part to cognitive impairment, lower awareness of risk, and challenges with infection control [5]. Once infected, individuals with schizophrenia have substantially higher rates of hospitalization and mortality [4, 6]. In a consecutive case series of 7,348 individuals with a positive COVID-19 laboratory test, Nemani, et al. [7] reported that history of a schizophrenia spectrum disorder diagnosis was associated with an increased odds of mortality (OR: 2.67; 95%CI: 1.48 – 4.80), an association not reported for either mood disorders or anxiety.

Studies in other populations report that risk factors for COVID-19 infection include demographic characteristics [8–10], social determinants [8, 9, 11, 12], psychiatric comorbidities [4, 8], and treatment [13, 14]. To date there have been no published reports of factors that increase the risk of infection among individuals with schizophrenia. The current research study builds on this preliminary evidence by evaluating the factors associated with risk of COVID-19 infection among a large, representative population of individuals with schizophrenia in the United States. It was the intent of this real-world, observational research study to identify risk factors that could be translated into actionable analytics to identify and target at-risk populations with outreach and interventions to reduce the risk of infection, reducing the consequences of COVID-19 in this vulnerable and under-served population.

## Methods

This study was a retrospective cohort study of 493,796 individuals who entered the COVID-19 pandemic with a diagnosis of schizophrenia. The study compared the population of individuals who did and did not receive a COVID-19 diagnosis by September 30, 2020. The study protocol was approved by the Advarra Institutional Review Board.

### Data sources and management

Individuals were identified and selected from medical and pharmacy claims Real-World Data licensed from Decision Resources Group. In addition, these claims data were used to measure healthcare resource utilization. Social determinants of health were measured at the county level from the Area Health Resources File available from the Department of Health and Human Services (www.data.hrsa.gov/topics/health-workforce/ahrf). Each individual was assigned to a county based on a combination of their 3-digit ZIP code, linked to the 5-digit ZIP code associated with their provider, assigned hierarchically beginning with the primary care provider, then the mental health care provider or clinic.

### Identification and selection of study participants

The study period was January 1, 2019 through September 30, 2020. Eligible individuals met each of the following criteria: diagnosis of schizophrenia or schizoaffective disorder (ICD-10-CM: F20.x, F25.x) defined by two or more outpatient claims (or one inpatient claim) between January 1, 2019 and June 30, 2020, 18 years of age or older as of January 1, 2019, a resident of the 50 United States or District of Columbia, and ongoing use of healthcare services as measured by at least one medical or pharmacy claim between each of January 1 and June 30, 2019; January 1 and June 30, 2020; and July 1 to September 30, 2020.

The index date for the pandemic was defined as March 1, 2020. The outcome measure, COVID-19 infection, was defined by ICD-10-CM B97.29 (from January 1 – March 31, 2020) and by U07.1 (from April 1 through September 30, 2020).

### Variable definitions

Covariates included demographic characteristics (age and gender), insurance type (Medicare, Medicaid, commercial, or VA/other), medical comorbidity measured by the Charlson Comorbidity Index [15] psychiatric comorbidities, health care resource utilization prior to the pandemic, and social determinants of health. Psychiatric comorbidities included anxiety (F41.x), adjustment disorders (F43.2x), bipolar depression (F30.x, F31.x), obsessive-compulsive disorder (F42.x), PTSD (F43.1x), sleep-wake disorders (G47.x), alcohol and substance use disorders (F10.x – F19.x, F32.x, F33.x, F34.1), and depressive disorders (F32.x, F33.x). Health care resource utilization was measured in the three-month period prior to March 1, 2020. Outpatient care was measured by an all-cause psychiatry office visit, psychotherapy session, or community mental health clinic visit. Acute care utilization was measured by partial-day hospitalization or schizophrenia-specific emergency department or inpatient hospitalization. Medication treatment was measured as at least one claim for an oral or long-acting injectable (LAI) antipsychotic. Social determinants of health, measured at the assigned county of residence included population density (residents per sq. mile as of 2010), median household income, education (% graduated high school), race (% African American/black, % white, % Asian), and ethnicity (% Hispanic).

### Statistical analysis

Frequencies and percentages were calculated for categorical variables: significance testing by chi-square test. Means and standard deviations for continuous variables: significance testing by two-sided T-test for mean. Adjusted odds ratios (OR) and 95% confidence intervals (CI) were derived from a multivariate logistic regression modeling of COVID-19 infection (dependent variable). The threshold for significance was set at 0.05. All analyses were performed with SAS software, v9.4, SAS Institute Inc., Cary, NC, USA.

## Results

The eligible study population of 493,796 individuals (Table 1) included residents of all 50 U.S. states and the District of Columbia, more males (55.2% vs. 44.8%), with an average age of 50.1 (SD: 15.5) years, and individuals primarily insured by Medicaid (68.2%) followed by Medicare (18.2%) and commercial plans (16.8%). The average Charlson Comorbidity Index score was 1.3 (SD: 1.9) and the most common diagnosed psychiatric comorbidity was anxiety disorder, affecting 31.5% of the population, followed by depressive disorders (29.7%), bipolar depression (24.5%), and sleep-wake disorders (16.7%). The most recent schizophrenia-associated diagnosis was unspecified schizophrenia (29.1%), bipolar-type schizoaffective disorder (25.5%), unspecified schizoaffective disorder (14.5%), paranoid schizophrenia (13.6%), and depressive type schizoaffective disorder (10.8%).

**Table 1:** Flow Diagram for the Selection of Eligible Participants

Step	Inclusion/Exclusion	Retained Cases	% Retained
1	≥ 2 diagnosis codes of schizophrenia from outpatient claims (at least one with schizophrenia diagnosis in primary position) OR one inpatient claim with schizophrenia in the primary position during the period January 1, 2019 through June 30, 2020.	710,948	100.0%
2	18 years of age or older as of January 1, 2019	704,552	99.1%
3	At least one medical or Rx claim in the first half of 2019 (Jan.~ Jun.) and at least one in the first half of 2020 (Jan.~ Jun.)	579,820	81.6%
4	Patients in 50 states and DC	578,011	81.3%
5	1+ Med or Rx claim in Q3, 2020	493,796	69.5%

In the three months prior to March 1, 2020, an estimated 24.8% and 6.5% of the population had a claim for an oral antipsychotic medication and an LAI antipsychotic, respectively. A total of 8.9% had a community mental health center visit, 8.3% had a psychiatry office visit, and 6.1% had psychotherapy session. Among the county-level social determinants of health, the average population density was 3,664 residents per square mile (SD: 9,914), the average median household income was $60,400 (SD: $15,800), with an average high school graduation rate of 87.0% (SD: 5.3%). The average county had 16.0% African American, 59.1% white, 16.4% Hispanic, and 5.7% Asian residents (Table 2).

**Table 2:** Select Demographic Characteristics

			Received COVID-19 Diagnosis				
	Total Population		No		Yes		
	*N*=	493796	*N*=	458547	*N*=	35249	
	n/(mean)	%/(SD)	n/(mean)	%/(SD)	n/(mean)	%/(SD)	*p*-value
**Demographics**							
Gender							
Female	221,217	44.8%	203,330	44.3%	17,887	50.7%	<0.001
Male	272,369	55.2%	255,007	55.6%	17,362	49.3%	
Unknown	210	0.0%	210	0.0%	0	0.0%	
Age							
18-30	66,513	13.5%	64,553	14.0%	1,960	5.6%	<0.001
31-49	158,898	32.3%	153,186	33.4%	5,712	16.2%	
50-64	176,889	35.8%	164,453	35.9%	12,436	35.3%	
Over 65	91,496	18.5%	76,355	16.7%	15,141	43.0%	
Insurance							
Commercial	66,630	13.5%	64,850	14.1%	1,780	5.0%	<0.001
Medicaid	336,542	68.2%	308,080	67.2%	28,462	80.7%	
Medicare	89,676	18.2%	84,766	18.5%	5,001	14.2%	
VA/Other	857	0.2%	851	0.2%	6	0.0%	
**Social Determinants**							
Income (median household income)	(60.43)	(15.79)	(60.37)	(15.77)	(61.24)	(15.98)	<0.001
Education							
% graduated high school	(87.0)	(5.3)	(87.1)	(5.3)	(86.5)	(5.4)	<0.001
Race							
African American/Black (%)	(16.0)	(14.1)	(16)	(14.1)	(16.6)	(13.6)	<0.001
White (%)	(59.1)	(22.2)	(59.3)	(22.2)	(56.6)	(22.2)	<0.001
Asian (%)	(5.7)	(6.1)	(5.7)	(6)	(6.2)	(6.4)	<0.001
Ethnicity							
Hispanic (%)	(16.4)	(15.9)	(16.3)	(15.9)	(18.1)	(16.6)	<0.001
Population Density (000s per sq mile)	(3664)	(9914)	(3637)	(9993)	(3550)	(8831)	<0.05
Charlson Comorbidity Index	(1.3)	(1.9)	(1.2)	(1.8)	(2.5)	(2.4)	<0.001
Behavioral, Psychiatric Comorbidities							
Anxiety disorder	155,636	31.5%	140,723	30.7%	14,913	42.3%	<0.001
Depressive disorders: MDD, dysthymia	146,549	29.7%	130,338	28.4%	16,256	46.1%	<0.001
Bipolar depression	121,164	24.5%	110,097	24.0%	11,067	31.4%	<0.001
Sleep-wake disorder	82,560	16.7%	73,975	16.1%	8,585	24.4%	<0.001
Alcohol Use disorder	59,207	12.0%	55,273	12.1%	3,934	11.2%	<0.001
Post-traumatic stress disorder	45,473	9.2%	43,023	9.4%	2,450	7.0%	<0.001
Adjustment disorders	16,879	3.4%	14,948	3.3%	1,931	5.5%	<0.001
Obsessive compulsive disorders	10,817	2.2%	10,012	2.2%	805	2.3%	NS
Baseline Treatment History							
Psychiatric visit (yes)	41,060	8.3%	39,733	8.7%	1,327	3.8%	<0.001
Psychotherapy visit (yes)	30,290	6.1%	29,237	6.4%	1,053	3.0%	<0.001
LAI use (yes)	32,338	6.5%	31,224	6.8%	1,114	3.2%	<0.01
Oral antipsychotic use (yes)	122,361	24.8%	117,013	25.5%	5,384	15.2%	<0.001
CMHC visit (yes)	43,741	8.9%	42,410	9.2%	1,331	3.8%	<0.001
Partial-day hospitalization visit (yes)	2,176	0.4%	2,052	0.5%	124	0.4%	<0.01
Hospitalization for schizophrenia (yes)	17,255	3.5%	15,982	3.5%	1,273	3.6%	NS
ED visit for schizophrenia (yes)	9,196	1.9%	8,485	1.9%	711	2.0%	<0.05

By September 30, 2020, a total of 35,249 (7.1%) individuals had received a COVID-19 diagnosis. Individuals diagnosed with COVID-19 were more likely to be female (50.7% vs. 44.3%), older (59.9 vs. 49.4 years of age), more likely insured by Medicaid (80.7% vs. 67.2%), and less likely insured by a commercial plan (5.0% vs. 14.1%) or Medicare (14.2% vs. 18.5%). Further, those with a COVID-19 diagnosis resided in counties with slightly higher income ($61.24K vs. $60.37K), lower rates of education (86.5% vs. 87.1%), and lower population density (3,550 vs. 3,673). Race and ethnicity also varied by county among those with and without a COVID-19 diagnosis, with positive cases residing in counties with a higher percent African American (16.6% vs. 16.0%), Hispanic (18.1% vs. 16.3%), and Asian (6.2% vs. 5.7%) residents. All comparisons were statistically significant.

COVID-19 positive individuals had higher Charlson Comorbidity Index (2.5 vs. 1.2) and were more likely to have anxiety disorder (42.3% vs. 30.7%), depressive disorders (46.1% vs. 28.4%), bipolar depression (31.4% vs. 24.0%), sleep-wake disorders (24.4% vs. 16.1%), and adjustment disorders (5.5% vs. 3.3%). However, infected individuals were less likely to be diagnosed with alcohol use disorder (11.2% vs. 12.1%) or post-traumatic stress disorder (7.0% vs. 9.4%).

Pre-pandemic health care utilization also varied between those with and without a COVID-19 diagnosis. Individuals who received a diagnosis were substantially less likely to have a pre-pandemic visit to a psychiatrist (3.8% vs. 8.7%), psychotherapist (3.0% vs. 6.4%), or community mental health center (3.8% vs. 9.2%) and to have pre-pandemic antipsychotic medication use: LAI antipsychotic (3.2% vs. 6.8%), oral antipsychotic use (15.2% vs. 25.5%). All results were statistically significant (Table 2).

### Regression

**Fig 1:**
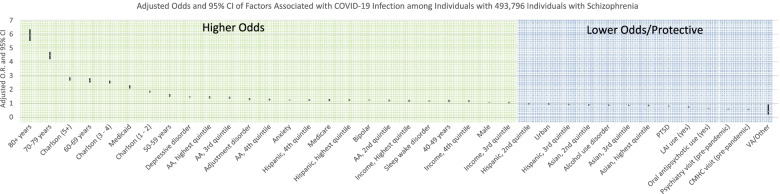
Adjusted Odds and 95% Confidence Inrtervals of Factors Associated with COVID-19 Infection among the 493,796 Individuals with Schizophrenia

With the exception of sex, the associations above remained after adjustment for all other factors. (Table 3; Figure 1) After adjustment, the factors most strongly positively-associated with COVID-19 infection were age (80+ years OR: 5.92, 70-79 years: OR 4.44, compared to 18-29 years), Charlson Comorbidity Index (5+ OR: 2.76; 3-4: 2.53; 1-2: 1.84), percent African American residents in the county (highest (5^th^) quintile: OR: 1.42, 4^th^ quintile: 1.26, 3^rd^ quintile: OR: 1.39; 2^nd^ quintile: 1.20), and select psychiatric comorbidities, with the strongest association with depressive disorder (OR: 1.45) followed by adjustment disorder (OR:1.29), anxiety (OR: 1.23), bipolar disorder (OR: 1.22), and sleep-wake disorder (OR:1.16). Factors strongly inversely-associated with COVID-19 infection were pre-pandemic utilization of healthcare services including a CMHC visit (OR: 0.55), psychiatry office visit (OR: 0.56), oral antipsychotic use (OR: 0.62), or LAI use (OR: 0.72), PTSD (OR 0.79) or alcohol use disorder (OR: 0.86), and higher percentage of Asian residents in a county (Table 3; Figure 1).

**Table 3:** Adjusted Odds Ratio and 95% Confidence Interval (CI) for COVID-19 Infection among Individuals with Schizophrenia

Variable	Category	Odds Ratio	95% CI	
Age	80+ years	5.92	5.51	6.35
	70-79 years	4.44	4.19	4.71
	60-69 years	2.65	2.51	2.80
	50-59 years	1.56	1.48	1.65
	40-49 years	1.16	1.09	1.23
	30-39 years	NS		
	18-29 years	1.00		
Sex	Male	1.08	1.05	1.10
	Female	1.00		
Insurer	VA/Other	0.41	0.18	0.91
	Medicare	1.23	1.16	1.30
	Medicaid	2.17	2.07	2.28
	Commercial	1.00		
Rural	Rural	NS	NS	NS
	Urban	0.94	0.90	0.99
	Metro	1.00		
Household Income	Highest quintile	1.17	1.11	1.23
	4th quintile	1.15	1.10	1.20
	3rd quintile	1.05	1.01	1.09
	2nd quintile	NS	NS	NS
	Lowest quintile	1.00		
Black/African American (%)	Highest quintile	1.42	1.35	1.49
	4th quintile	1.26	1.20	1.32
	3rd quintile	1.39	1.33	1.46
	2nd quintile	1.20	1.15	1.26
	Lowest quintile	1.00		
Asian (%)	Highest quintile	0.83	0.78	0.88
	4th quintile	NS	NS	NS
	3rd quintile	0.84	0.80	0.88
	2nd quintile	0.87	0.83	0.92
	Lowest quintile	1.00		
Hispanic (%)	Highest quintile	1.23	1.17	1.28
	4th quintile	1.23	1.18	1.28
	3rd quintile	0.90	0.87	0.94
	2nd quintile	0.96	0.92	1.00
	Lowest quintile	1.00		
Charlson Comorbidity Index	5+	2.76	2.65	2.87
	3 - 4	2.53	2.44	2.63
	1 - 2	1.84	1.78	1.90
	0	1.00		
Psychiatric Comorbidities	Depressive disorder	1.45	1.41	1.49
	Adjustment disorder	1.29	1.23	1.36
	Bipolar	1.22	1.19	1.25
	Anxiety	1.23	1.20	1.26
	Sleep wake disorder	1.16	1.13	1.19
	Alcohol use disorder	0.86	0.82	0.89
	PTSD	0.79	0.75	0.82
Psychiatric Care	Psychiatry visit (pre-pandemic)	0.56	0.53	0.59
	CMHC visit (pre-pandemic)	0.55	0.52	0.58
	LAI use (yes)	0.72	0.68	0.77
	Oral antipsychotic use (yes)	0.62	0.60	0.64

## Discussion

By the end of September 2020, the estimated prevalence rate of COVID-19 infection among the study population was 7.1%, a rate more than three times higher than the general population, with 7,262,734 (2.2%) Americans infected by COVID-19 (NY Times COVID Data, 2021) among the 332.6M Americans estimated by the U.S. Census Bureau (U.S. Census, 4/1/20). Though our study was not designed to estimate increased risk of infection among individuals with schizophrenia, elevated risk of infection among individuals with schizophrenia has been reported by others [8, 16, 17].

Among our study population, social determinants of health were significant contributors to individual risk of infection, with higher rates realized by individuals insured by Medicaid or who lived in counties with higher proportions of African American or Hispanic residents and higher rates among individuals residing in higher income areas. We identified three studies in general populations that reported similar results. In a retrospective analysis of COVID-19 infection among 34,503 individuals who sought care at a single regional health system, Rozenfeld [9] reported adjusted odds ratios of 1.51 for African Americans and 2.07 among individuals of Latino ethnicity. Lan [11] reported COVID-19 incidence rate ratios of 2.78 and 2.41 among African American and Hispanic healthcare workers, compared to non-Hispanic white colleagues and in a state-level analysis, Padalabalanarayanan [12] estimated 4.6% more COVID-19 cases for each percent increase in a state’s African American population. In one of the only other studies to report on individuals with schizophrenia, Wang [8] reported an adjusted odds of infection of 2.3 among African Americans (compared to Caucasians) with schizophrenia. The consistently elevated risk of infection among African Americans and individuals in low-income household is particularly concerning because these populations are at increased risk for poorer outcomes following COVID-19 infection [8, 18, 19].

Further, after accounting for demographic and social determinants, comorbid depressive disorder, bipolar disorder, adjustment disorder, anxiety, and sleep-wake disorders were each independently associated with increased risk of COVID-19 infection. This is consistent with the findings of Taquet [17], who reported that the hazard ratio for a diagnosis of COVID-19 infection was greatest for anxiety disorders, insomnia, and dementia and with those of Wang [8], who reported elevated adjusted odds of COVID-19 infection among individuals with ADHD (adjusted OR: 7.3), bipolar disorder (OR: 7.7), depression (OR: 10.4), and schizophrenia (OR: 9.9).

In contrast, a diagnosis of either alcohol use disorder or post-traumatic stress disorder (PTSD) was associated with lower odds of COVID-19 infection. The reduced risk of COVID-19 infection among individuals with alcohol use disorder is consistent with the results of Rozenfeld [9] who reported individuals with substance use disorder had reduced risk of infection and with Yazdi [20], who reported that 43% of alcohol use disorder patients were more like to live alone and have reduced social interactions, leading to a decreased infection risk. COVID-19 infection as a risk factor for PTSD has been well studied [21–23]. Less research has been published on pre-existing PTSD as a risk factor for COVID-19 infection. In a systematic review and meta-analysis of mental health and neurological disorder predictors of COVID-19 infection, Liu, et al. [24] identified a total of 149 eligible manuscripts, only two of which included “stress-related disorders” as a predictive factor. In contrast to pre-existing mental health diagnosis of ADHD, anxiety, mood disorders, non-specific mental disorders, and any mental disorder each of which increased risk of COVID-19 infection, pre-existing stress disorder was deemed non-significant (OR: 1.05; 95% CI 0.67 – 1.64). In a study among veterans, Haderlein, et al., [25] reported that veterans with clinically-diagnosed PTSD were both more likely to be tested for COVID-19 infection and less likely to test positive for COVID-19. The report indicated that the lower risk of infection may be an artefact of higher testing rates or a true measure of lower risk associated with elevated concern of infection and with the social isolation common in this population.

Consistent with the increased risk among older adults, individuals with higher comorbidity burden were also at increased risk of infection. This may be a consequence of poorer health, compromised immune function, greater healthcare needs, and the inability to maintain physical distancing [19]. Bitan et al. [6] reported that physical health comorbidities were associated with higher COVID-19 mortality rates, as schizophrenia patients were more likely to be obese, smoke, and be diagnosed with diabetes and COPD.

Among the most striking results, individuals with schizophrenia who were actively receiving mental health care in the three months prior to March 2020 had dramatically lower risk of COVID-19 infection. Individuals with a recent, pre-pandemic psychiatry visit, psychotherapy session or community mental health center visit were substantially less likely to receive a COVID-19 diagnosis. These results suggest that active management of schizophrenia reduces the behaviors that lead to exposure and improves patient awareness of the risks associated with COVID-19 [26].

Though the inverse association between COVID-19 infection and antipsychotic medication use documented in our study may also represent the value of active therapeutic management, other research conducted to date has yielded inconsistent and conflicting results about the association between antipsychotic treatment and COVID-19 infection risk. Recently published studies have documented both an increased risk of infection [14] and a decreased risk of infection among patients on antipsychotics [13]. The inflammatory mechanisms involved in psychiatric illness and COVID-19 infection in combination with the anti-inflammatory and antiviral effects of antipsychotics appear to be a key driver of the ongoing research [27]. As an example, Crespo-Facorro [28] reported that aripiprazole and COVID-19 modulate the expression of genes that modulate immune and inflammatory response at a rate substantially higher than expected.

### Strengths and limitations

The study included several strengths and limitations. The primary strength is that the study population of 493,796 individuals, represents a substantial proportion of all individuals with diagnosed schizophrenia in the United States, increasing the generalizability of results. Second, the population included individuals across a spectrum of ages, insurance types, and geographic regions. Third, the study included both healthcare utilization and social determinants of health data. The most significant limitation is the open dataset, which does not include insurance eligibility information and thus may result in incomplete ascertainment of healthcare utilization. The second substantive limitation is that social determinants are measured at the county, rather than individual, level and thus may not represent an individual’s specific situation. Further, the populations with schizophrenia and schizoaffective disorder were not analyzed separately. Finally, the dataset includes neither symptom severity nor mortality information.

## Conclusions

In the general U.S. population, individuals diagnosed with schizophrenia were at significantly higher risk of COVID-19 infection and our study suggests that risk was elevated further among the underserved, among African Americans and the Hispanic population, and among those with psychiatric comorbidity. The protective effect of psychiatric care and of antipsychotic medication use underscored the importance of continual care throughout the pandemic.

## Data Availability

The administrative medical and pharmacy claims data that support the findings of this study are the Real World Data available from Clarivate (https://clarivate.com/products/real-world-data/) but restrictions apply to the availability of these data, which were used under license for the current study, and so are not publicly available. Data may be available from the authors upon reasonable request and with written permission of Clarivate. The social determinants of health data are publicly available as part of the Area Health Resources Files, available at https://data.hrsa.gov/topics/health-workforce/ahrf.

## Abbreviations

CI: Confidence Interval
CMHC: Community Mental Health Center
COPD: Chronic Obstructive Pulmonary Disease
ICD-10: International Classification of Diseases, Tenth Revision
LAI: Long-Acting Injectable
OR: Odd Ratio
PTSD: Post-Traumatic Stress Disorder
SD: Standard Deviation
